# P-1463. Effects of recombinant shingles vaccination on incident dementia among newly-admitted US nursing home residents: a target trial emulation

**DOI:** 10.1093/ofid/ofaf695.1649

**Published:** 2026-01-11

**Authors:** Kaleen Hayes, Daniel Harris, Kevin McConeghy, Lexie Grove, Richa Joshi, Preeti Chachlani, H Edward Davidson, Lisa Han, Peyton Free, Mriganka Singh, Thomas Bayer, Yasin Abul, Stefan Gravenstein

**Affiliations:** Brown University, Providence, Rhode Island; University of Delaware, Newark, Delaware; COIN-LTSS, Providence Veterans Affairs Medical Center, Providence, Rhode Island; Brown University School of Public Health, Takoma Park, Maryland; Center for Gerontology and Healthcare Research, Brown University School of Public Health, Providence, Rhode Island; Center for Gerontology and Healthcare Research, Brown University, Bellingham, Massachusetts; Insight Therapeutics LLC, Norfolk, Virginia; Insight Therapeutics, LLC, Norfolk, Virginia; University of Delaware, Newark, Delaware; Alpert Medical School, Providence, Rhode Island; Providence VA Medical Center, Providence, Rhode Island; Brown University, Providence, Rhode Island; Brown University, Providence, Rhode Island

## Abstract

**Background:**

Several ecological studies have shown a protective association between herpes zoster vaccination and dementia risk, yet these studies have weaknesses that limit causal inferences. We emulated a randomized trial in observational data using the clone-censor weight method to estimate the effects of a recombinant herpes zoster vaccination (RZV) strategy on dementia incidence among newly admitted nursing home (NH) residents.

**Methods:**

We used NH electronic health record (EHR) data linked to Medicare claims to identify newly admitted residents between 01/01/2017-12/31/2022. As of the first admission assessment date in the NH, we applied all eligibility criteria (≥ 66 years of age, enrolled in Medicare Fee-for-Service and Part D for 12 months, eligible to receive RZV, and without dementia), “cloned” eligible residents (i.e., duplicated each record in the dataset), and "assigned" each clone to receive one of two exposures to vaccination: 1) receive at least one RZV dose within one year or 2) do not receive RZV. Clones were followed for up to four years to capture incident dementia diagnoses (primary outcome) and censored upon non-adherence to their assigned treatment strategy, Medicare disenrollment, or death, whichever occurred first. Pooled logistic regression compared the risk of dementia between vaccine treatment strategies. Models were adjusted using inverse probability of censor weights that included baseline clinical and demographic covariates.

**Results:**

We identified 509,926 NH residents (mean age 79.3 (SD=8.2) years; 63.6% female). Among those still alive, uncensored, and without dementia at 12 months, 8,774 received at least one dose of RZV (60% received dose two) and 256,031 were unvaccinated. Receipt of at least one dose of RZV within one year of NH admission was associated with a 6.0% absolute risk reduction (95%CI: 7.8%-3.7% lower) of dementia over follow-up (cumulative incidence of 19.6% in the RZV group versus 25.7% in the no RZV group; risk ratio = 0.76 [95%CI: 0.70-0.85]).

**Conclusion:**

Administering RZV within one year of nursing home admission may reduce the risk of incident dementia, yet additional adjustment for time-varying confounding and competing risk are needed to confirm the direction and magnitude of the effect size.

**Disclosures:**

Kaleen Hayes, PharmD, PhD, Genentech: Grant/Research Support|GlaxoSmithKline: Grant/Research Support|Sanofi: Grant/Research Support Daniel Harris, PhD, MPH, GSK: Grant/Research Support|Insight Therapeutics: Advisor/Consultant|Sanofi: Advisor/Consultant Kevin McConeghy, Pharm.D., GlaxoSmithKline: Investigator-Initiated Study|Moderna: Grant/Research Support H Edward Davidson, PharmD, GSK: Grant/Research Support|Moderna: Grant/Research Support|Sumitomo: Grant/Research Support Lisa Han, MPH, GSK: Grant/Research Support|Moderna: Grant/Research Support|Sumitomo: Grant/Research Support Mriganka Singh, MD, Glaxo Smith Kline: Grant/Research Support Yasin Abul, MD, CDC/ABT: Grant/Research Support|CLARIO: Advisor/Consultant|GSK: Grant/Research Support|Moderna: Grant/Research Support|Seqirus: Grant/Research Support Stefan Gravenstein, MD, MPH, GSK: Advisor/Consultant|GSK: Grant/Research Support|GSK: Honoraria|Moderna: Grant/Research Support|Novavax: Advisor/Consultant|Novavax: Honoraria|Pfizer: Advisor/Consultant|Pfizer: Grant/Research Support|Pfizer: Honoraria|Sanofi: Advisor/Consultant|Sanofi: Grant/Research Support|Sanofi: Honoraria|Seqirus: Grant/Research Support

